# High flow oxygen therapy versus conventional oxygen therapy in dogs and cats undergoing bronchoscopy and bronchoalveolar lavage: a pilot study

**DOI:** 10.3389/fvets.2024.1360017

**Published:** 2024-05-24

**Authors:** Camille Dartencet, Maha Abunemeh, Stephane Junot, Alexandra Nectoux, Bernard Allaouchiche, Emilie Krafft, Celine Pouzot-Nevoret

**Affiliations:** ^1^Intensive Care Unit (SIAMU), Université de Lyon, VetAgro Sup, Marcy l'Etoile, France; ^2^APCSe, Université de Lyon, VetAgro Sup, Marcy l'Étoile, France; ^3^Department of Veterinary Anesthesia and Analgesia, Université de Lyon, VetAgro Sup, Marcy l'Étoile, France; ^4^Hospices Civils de Lyon, Centre Hospitalier Lyon-Sud, Service de Réanimation, Pierre-Bénite, France; ^5^Small animal medicine, USC1233 RS2GP, INRAe, Université de Lyon, VetAgro Sup, Marcy l'Étoile, France

**Keywords:** high flow oxygen, bronchoscopy, hypoxemia, dogs, cats

## Abstract

**Objectives:**

To evaluate the safety and feasibility of high flow oxygen therapy (HFOT), and to record SpO_2_ and desaturation episodes in dogs and cats receiving HFOT or conventional oxygen therapy (COT) during bronchoscopy ± bronchoalveolar lavage (BAL).

**Materials and methods:**

Dogs and cats undergoing bronchoscopy ± BAL between January and May 2023 were included in the study. Patients were randomly allocated to two groups: HFOT (HFOT group; two cats and four dogs) and COT (COT group; one cat and five dogs). HFOT and COT were started at the beginning of the bronchoscopy. HFOT was delivered with a gas flow rate of 1 L/kg/min at an FiO_2_ of 100% and a temperature of 34°C (pediatric mode) or 37°C (adult mode). COT was delivered through the working channel of the bronchoscope at a rate of 1.5 L/min. The safety and feasibility of HFOT were assessed, and peripheral oxygen saturation (SpO_2_) was measured by pulse oximetry every 30 s throughout the procedure.

**Measurements and main results:**

HFOT was feasible and safe in both dogs and cats with no complications reported. While there was no significant difference in the number of desaturation episodes (SpO_2_ < 94%) between the two groups, none of the patients in the HFOT group experienced severe desaturation (SpO_2_ < 90%). In contrast, two patients in the COT group had an SpO_2_ < 90%. Mean SpO_2_ was significantly higher in the HFOT group compared to the COT group at T0 (98% ± 2% vs. 94 ± 2%), T0.5 (98% ± 2% vs. 94% ± 3%) and T1 (98% ± 2% vs. 94% ± 4%).

**Conclusion:**

To the authors’ knowledge, this is the largest study conducted to date using HFOT during bronchoscopy in dogs and cats. Our results suggest that HFOT is feasible and safe during bronchoscopy ± BAL. Furthermore, HFOT may reduce the risk of desaturation episodes in dogs and cats undergoing bronchoscopy and BAL.

## 1 Introduction

High flow oxygen therapy (HFOT) is a non-invasive technique that provides humidified and heated gas with a specific oxygen concentration [fraction of inspired oxygen (FiO_2_)] (1). The HFOT device comprises an adapted high flow nasal cannula that allows the operator to choose the temperature (from 31°C to 37°C), the FiO_2_ (from 21 to 100%) and the flow rate (up to 70 L/min) of the delivered gas. In recent years, HFOT has been increasingly used in veterinary medicine. This technique can improve oxygenation parameters and reduce work of breathing in both healthy (2) and dyspneic dogs (3–6). Furthermore, it has been used successfully in dogs with upper airway obstruction (7). Only one study reported a complication (persistent pneumothorax) secondary to the use of HFOT (3). No other study have reported major complications secondary to the use of HFOT in veterinary patients (2, 4–7).

Tracheobronchoscopy employs a flexible bronchoscope to visualize the mucosal surfaces of the trachea and lobar bronchi (8). This procedure allows bronchoalveolar lavage (BAL) to be performed under visual guidance. Bronchospasm and oxygen desaturation episodes are well-known complications of tracheobronchoscopy (8). In a study of perianesthetic complications in cats undergoing bronchoscopy, almost one third of cats experienced an SpO_2_ less than 90% (9).

Desaturation episodes also occur in human patients undergoing bronchoscopy. Therefore, several human studies have assessed whether HFOT can improve oxygenation during bronchoscopy. In a prospective randomized study, arterial partial pressure of oxygen (PaO_2_), PaO_2_/FiO_2_ ratio and SpO_2_ were higher in patients treated with HFOT during bronchoscopy compared with patients oxygenated with a venturi mask (10). In another recent study evaluating patients at high risk for hypoxemia, HFOT during bronchoscopy was associated with a lower number of desaturation episodes and a reduction in the need for thrust maneuvers, compared with conventional oxygen therapy (COT) (11). High flow oxygen therapy is also associated with a reduced risk of desaturation episodes in children during bronchoscopy and BAL compared with COT (12). Furthermore, several systematic reviews and meta-analyses have concluded that HFOT is superior to COT during bronchoscopy in both healthy and high-risk hypoxemic adults (13–17).

To the best of our knowledge, there is only one published veterinary study assessing the feasibility of HFOT during bronchoscopy. That study was uncontrolled and only included four dogs (18). Therefore, the aims of this pilot study were, in dogs and cats undergoing bronchoscopy ± BAL, to evaluate the safety and feasibility of HFOT and to compare pulse oxymetry and the number of desaturation episodes with HFOT and COT. We hypothesized that HFOT would be safe and feasible during bronchoscopy. We also hypothesized that HFOT would reduce the frequency and severity of desaturation episodes during bronchoscopy compared to COT.

## 2 Materials and methods

### 2.1 Ethical statement

The study protocol was approved by the VetAgro Sup Ethics Committee (approval number: 2312).

### 2.2 Animals

This prospective pilot study was conducted between January and May 2023 at the veterinary teaching hospital of VetAgro Sup – Campus Vétérinaire de Lyon. All dogs and cats undergoing diagnostic bronchoscopy for acute or chronic respiratory disease were eligible for inclusion. The decision to perform bronchoscopy was made by the attending internal medicine clinician and informed written consent was obtained from the owner prior to enrollment of each patient. Exclusion criteria included the following conditions: complete obstructive nasal disease preventing the use of high flow nasal cannulas; upper airway leak (e.g., tracheal tear, tracheostomy tube); pneumothorax (confirmed by point-of-care ultrasound or chest radiograph); and suspected increased intracranial pressure (decreased alertness, bradycardia, and systemic hypertension).

After enrollment, dogs and cats were randomized into two groups using a web-based randomization software (random function (0;1) in Excel, Microsoft): animals assigned to the HFOT group received HFOT via nasal cannulas and animals in the COT group received oxygenation through the working channel of the bronchoscope. High flow oxygen therapy was discontinued immediately if epistaxis or gastric dilation was detected during the procedure.

### 2.3 Anesthesia

Patient information including age, sex, breed, weight, reason(s) for bronchoscopy, and comorbidities were recorded. Each patient was monitored throughout the procedure by an emergency and critical care (ECC) clinician (CD) or an anesthetist (MA or SJ).

Prior to anesthesia, each animal underwent physical examination and intravenous catheterization. A baseline dyspnea score (DS pre) [Table 1, (7)] and SpO_2_ measurement were obtained by the ECC clinician. The internal medicine clinician made a subjective assessment of the risk of desaturation. The risk level was classified as either usual or high based on species, medical history, physical examination at enrollment, hypotheses regarding underlying respiratory disease, and results of additional testing (i.e., thoracic imaging, arterial blood gas). Dyspnea score (DS post), SpO_2_ measurement, and complete physical examination were repeated at the end of bronchoscopy.

**Table 1:** Dyspnea score [adapted from Jagodich et al. (5, 7)].

Score	Parameters
0	Normal respiratory rate (< 40), no effort
1	Respiratory rate ∼40–48, no use of accessory muscles
2	Respiratory rate ∼40–48 and/or mild focused on respirations, mild abdominal component to breathing, occasionally will lie down
3	Respiratory rate 48–60 and/or moderate respiratory effort
4	Respiratory rate > 60 and/or marked respiratory effort

All patients were premedicated with butorphanol (Torbugesic, 10 mg/mL, Zoetis) and dexmedetomidine (Dexdomitor, 0.5 mg/mL, Vetoquinol S.A.) and preoxygenated with a face mask for 2 min. Each cat received one dose of inhaled salbutamol (Ventoline, 100 μg/dose, Pfizer) via an aerosol holding chamber (Aerokat, Trudell Medical International) immediately after the premedication. All patients were anesthetized using a standardized total intravenous protocol, which consisted of an initial intravenous bolus of propofol (PropoVet, 10 mg/mL, Zoetis) to obtain narcosis, followed by a continuous rate infusion of 0.1–0.4 mg/kg/min. Any deviation from this protocol was recorded.

### 2.4 High flow oxygen therapy group (HFOT group)

**Figure 1:**
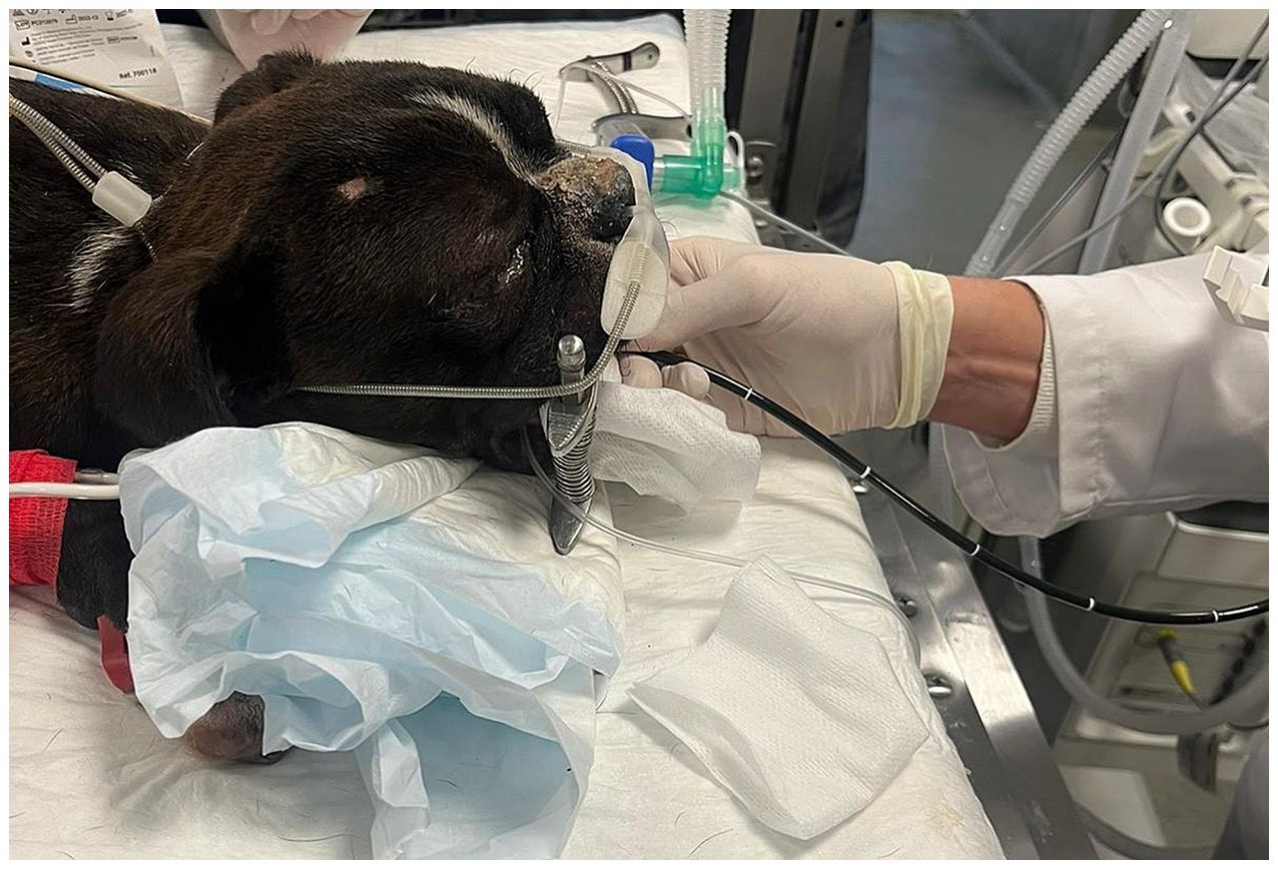
Dog undergoing bronchoscopy with high flow oxygen therapy.

High flow oxygen therapy was delivered using the AirvoTM 2 system (Fisher-Paykel AirvoTM 2 system, Fisher & Paykel Healthcare) in the pediatric mode for gas flow rates below 25 L/min or in the adult mode for gas flow rates above 25 L/min, with bilateral soft silicone nasal cannulas as the patient interface (Figure 1). Prong size was selected to occupy approximately 50% of the nares diameter. During the premedication time period, the ECC clinician (CD) set the HFOT device using a gas flow rate of 1 L/kg/min delivered through the HFOT device at an FiO_2_ of 100% and a temperature of 34°C (pediatric mode) or 37°C (adult mode). The flow rate was rounded up or down to the nearest 0.5 L to a non-decimal value and started upon insertion of the bronchoscope into the mouth. Nasal cannulas were placed by the ECC clinician (CD) into the nostrils after induction of anesthesia After bronchoscopy, HFOT was continued until SpO_2_ was >95%.

### 2.5 Conventional oxygen therapy group (COT group)

During bronchoscopy, oxygen was delivered through the working channel of the bronchoscope at a rate of 1.5 L/min. This rate was arbitrarily chosen to reflect a potential flow-by rate and because it produced a flow that could be felt at the end of the bronchoscope. Oxygen delivery was paused during BAL and then resumed until the end of the procedure. After bronchoscopy, O_2_ was delivered by flow-by and stopped when SpO_2_ > 95%.

### 2.6 Study protocol

All bronchoscopies were performed by an internal medicine resident under the supervision of a board-certified internal medicine specialist or French national internal medicine specialist (DESV).

Pulse oximetry was recorded every 30 s throughout the procedure. Time was recorded as follows: T0 (start of bronchoscopy); T0.5 (30 s after the start of bronchoscopy); T1 (1 min after the start), T1.5 (1 min and 30 s after the start), and so on. Vital parameters including respiratory rate, heart rate, temperature, and non-invasive arterial blood pressure were continuously monitored with a multiparameter monitor (iPM12 Vet Monitor, Mindray) and recorded every 5 min. Pulse oxymetry values were collected until the end of bronchoscopy and vital parameters were recorded until full anesthetic recovery.

A desaturation episode was defined as an SpO_2_ < 94%, and severe desaturation as an SpO_2_ < 90%. If severe desaturation occurred, bronchoscopy was interrupted for intubation and ventilation with 100% oxygen. The number of intubations and the reason for each intubation was recorded. When SpO_2_ returned to 95%, the animal was extubated and the bronchoscopy was resumed.

Bronchoscopy was performed under general anesthesia in sternal recumbency with either a 4.2 mm flexible video bronchoscope (Olympus EVIS EXERA III Video bronchoscope BF-P190N, 2 mm working channel) or a 5.8 mm flexible video gastroscope (Olympus EVIS EXERA III Video gastroscope GIF-XP190N, 2.2 mm working channel). Bronchoalveolar lavage was performed during bronchoscopy as follows: warm sterile saline solution (NaCl 0.9%) was instilled with a syringe through the working channel into at least 2 different lung lobes (in cats, 2 aliquots of 5 to 10 mL into each lung lobe; in dogs, 2 to 3 aliquots of 1 mL/kg into each lung lobe) and either withdrawn into the same syringe by gentle hand suction or collected by low-power pump aspiration into a single sterile container. The amount of fluid injected and collected during the lavage was recorded, along with bronchoscopic findings, BAL fluid cytology, and culture results. The total duration of the procedure (bronchoscopy with or without BAL) was recorded in minutes. Beginning of bronchoscopy was defined as when the bronchoscope passed the larynx. Patients were monitored for potential complications associated with HFOT, such as pneumothorax, marked aerophagia (defined as abdominal distension with significant tympany and visible gas-filled stomach on point-of-care ultrasound or abdominal radiographs if performed), onset of epistaxis, and hyperthermia (rectal temperature above 39.1°C). All complications were recorded.

### 2.7 Outcomes

Primary outcomes were the feasibility and safety of HFOT during bronchoscopy, specifically the time and ease of device set up, bronchoscopy operator interference, and technique-related complications. Secondary outcomes included assessment of SpO_2_ and the number of desaturation episodes and severe desaturation episodes during the procedure.

### 2.8 Statistical analysis

Descriptive statistics were used to describe signalment, clinical signs, desaturation risk and diagnosis. Normality of continuous variables was tested using the Shapiro–Wilk test. Age, respiratory rate, heart rate, temperature, and SpO_2_ followed a normal distribution and were expressed as mean (± standard deviation). Other continuous variables were expressed as median (Q1; Q3) where Q1 was the first quartile and Q3 was the third quartile. Categorical variables were expressed as number and frequency.

A Student’s *t*-test was used for continuous normally distributed variables and a Mann–Whitney test was used for other continuous variables. For categorical variables, a chi-squared test was used when the assumptions were valid (expected frequencies >5 in each subgroup). Otherwise, a Fisher’s exact test was used. Pulse oximetry variations were analyzed using a mixed model with oxygenation mode as a fixed factor and time and subject as random factors (JMP®, Version 17.1 SAS Institute Inc., Cary, NC, 1989–2023). We defined the overall SpO_2_ as the mean pulse oximetry of the patients (depending on the group) across the study period. The between-group variation was calculated by comparing the mean of each group with the overall mean of the data.

The alpha risk was set at 5 %, meaning that a *p*-value less than 0.05 was considered a significant difference.

Due to the design of this study, power analysis could not be performed. The study was therefore not designed to detect significant differences.

## 3 Results

### 3.1 Cohort population

Twelve patients, including nine dogs and three cats, were enrolled. Patient characteristics are shown in Table 2. The dog population included two pugs, two Australian shepherds, one husky, one French bulldog, one English bulldog, one Yorkshire terrier, and one white American shepherd. All cats were domestic shorthair. Six patients (one cat and five dogs) were randomly assigned to the COT group and six (two cats and four dogs) to the HFOT group. Mean age (4 ± 4 years and 6 ± 3.8 years, respectively, *p* = 0.35) neither median weight (6.5 (4.8; 16.7) kg and 10 (6.35; 28) kg, respectively, *p* = 0.40) were not significantly different between the HFOT group and the COT group. All six dogs and one of the three cats were considered at having a usual risk of desaturation. Two cats with suspected lower airway disease were at high risk of desaturation. These two cats were assigned to the HFOT group.

**Table 2:** Characteristics of patients data in the conventional oxygen therapy (COT) and high flow oxygen therapy (HFOT) groups (BOAS: brachycephalic obstructive airway syndrome, DSH: Domestic shorthair, W: weight) with age in years and weight in kilograms (kg).

	Case	Specie	Breed	Age	W	Diagnosis
COT	1	Dog	American white sheperd	12	28	Laryngeal paralysis, severe laryngitis
	2	Dog	French bulldog	1	10	BOAS
	3	Dog	English bulldog	4	28	BOAS
	4	Dog	Pug	5	9	BOAS
	8	Dog	Australian sheperd	8	36.6	Chronic bronchitis
	10	Cat	DSH	8	3.7	Severe hyperplasic laryngitis
HFOT	5	Dog	Pug	0.5	5	BOAS
	6	Dog	Australian sheperd	1	16	Chronic bordetellosis
	7	Dog	Husky	4	19	Eosinophilic bronchopneumopathy
	9	Dog	Yorkshire	8	8	Tracheal collapse, segmental bronchomalacia, multifocal bronchiectasia
	11	Cat	DSH	10	5.1	High suspicion of diffuse lung carcinoma plus congenital partial choanal atresia
	12	Cat	DSH	1	4.4	Chronic bronchitis

One cat (HFOT group) required an intramuscular injection of alfaxalone (Alfaxan, 10 mg/mL, Jurox) during premedication. In addition, one dog (HFOT group) with a known cardiomyopathy did not receive dexmedetomidine. Brachycephalic dogs undergoing exploration and surgery for brachycephalic obstructive airway syndrome were administered methadone (Confortan, 10 mg/mL, Eurovet Animal Health) at 0.2 mg/kg instead of butorphanol during premedication. All other patients received the anesthetic protocol described in the Materials and Methods section.

No complications associated with HFOT were recorded during the study period. Subjective assessment by the operators indicated that the HFOT device was quick and easy to set up, and that the nasal cannulas did not interfere with the bronchoscopic procedure. All dogs and cats received the flow and FiO_2_ outlined in the study design.

### 3.2 Physiologic parameters during bronchoscopy

Table 3 shows the clinical data before and after the procedure, the duration of bronchoscopy, whether BAL was performed, and BAL yield for all patients included in the study. Dyspnea scores and BAL yield for case 8 were missing, and the post-procedure temperature measurement was not available for case 7. Vital parameters were within the normal range for all patients except for the two cats in the HFOT group that exhibited tachypnea.

**Table 3:** Characteristics of patients in the conventional oxygen therapy (COT) and high flow oxygen therapy (HFOT) groups (BAL: bronchoalveolar lavage, DS: dyspnea score, RR: respiratory rate, HR: heart rate, T: temperature, pre: before the bronchoscopy, post: after the bronchoscopy, P: panting, −: data not collected, /: not applicable) with time in minute, SpO_2_ in percentage, heart rate in beats per minute, respiratory rate in breaths per minute, temperature in Celsius degrees and yield in percentage.

	Case	DS pre	DS post	SpO_2_ pre	SpO_2_ post	HR pre	HR post	RR pre	RR post	T pre	T post	Time	Time BAL	BAL yield	Intubation
COT	1	2	1	98	98	90	120	36	40	38.5	38.9	2	/	/	Yes
	2	0	0	100	100	100	60	40	13	38.7	37.3	3	/	/	No
	3	2	0	98	98	120	60	P	20	39	38.3	5	/	/	No
	4	0	0	96	100	120	60	20	20	37.8	38	2	/	/	No
	8	–	–	97	93	116	66	P	88	38.7	38.5	5	2	–	No
	10	4	4	100	92	134	140	28	36	37.4	37.4	2	/	/	Yes
HFOT	5	0	0	95	95	80	60	40	20	38.2	37.1	3	/	/	No
	6	0	0	98	95	72	54	32	28	38.5	38.9	18	9	45	No
	7	0	0	97	100	80	60	P	20	38.7	-	9	5	67	No
	9	0	2	96	94	90	60	40	36	37.8	38	10	4	20	No
	11	3	3	100	96	180	160	60	40	37.7	37.9	28	15	60	No
	12	1	1	97	99	202	140	70	60	38.3	38.2	3	1	60	No

Tracheobronchoscopy was performed with a video gastroscope in cases 1, 3, 4, 5, 7, and 8 and with a video bronchoscope in cases 2, 6, 9, 10, 11, and 12.

**Figure 2:**
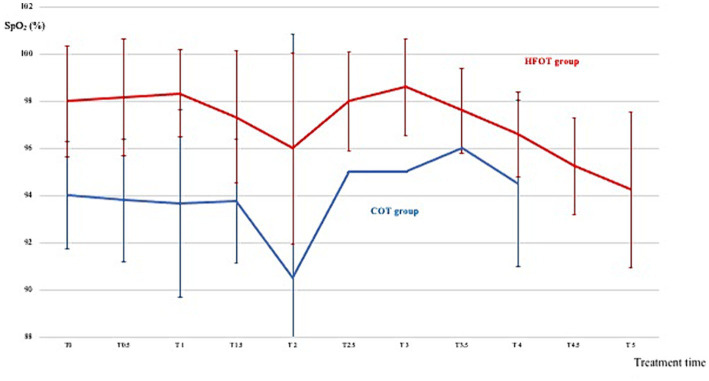
Mean pulse oxymetry during the first 5 min of bronchoscopy in the conventional oxygen therapy (COT) group (blue line) and the high flow oxygen therapy (HFOT) group (red line). Vertical bars represent the standard deviation.

**Figure 3:**
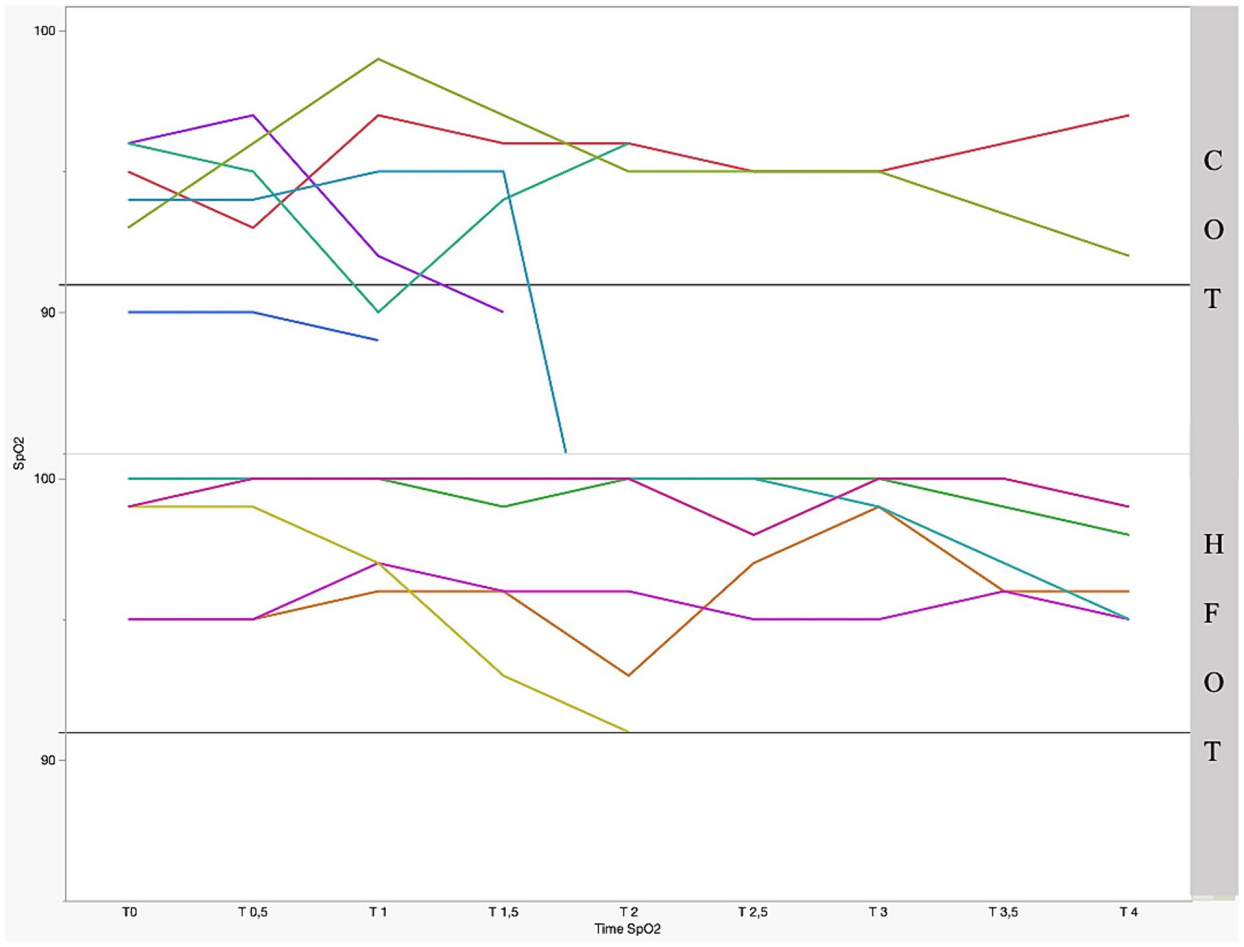
Individual pulse oxymetry (SpO_2_) during bronchoscopy as a function of time and group with significant difference between the two groups (*p* = 0.01); the straight gray line represents a threshold for severe desaturation at an SpO_2_ = 91%. Each colored line represents 1 patient.

Figure 2 shows the mean SpO_2_ and Figure 3 shows individual SpO_2_ over time in the COT and HFOT groups. The bronchoscopy procedure was terminated prematurely in two patients in the COT group due to severe desaturation. Severe desaturation was not recorded in any of the patients in the HFOT group.

An increase in the dyspnea score was observed in 1/6 patients receiving HFOT and in none of the patients in the COT group (Table 3). This dog had a dyspnea score of 0 before and 2 after the bronchoscopy. This patient had the lowest BAL yield (20%) and exhibited a desaturation episode after the BAL procedure. The dog was weaned from HFOT at the end of the BAL, but required supplemental flow-by oxygen for 30 min until full recovery.

### 3.3 Univariate analysis

There was no difference in dyspnea scores between the groups before and after bronchoscopy (Table 4). The median duration of bronchoscopy in the HFOT group [9.5 (3; 20.5) minutes] was significantly longer than in the COT group [2.5 (2; 5) minutes, *p* = 0.04, Table 4].

**Table 4:** Univariate analysis between the conventional oxygen therapy (COT) and high flow oxygen therapy (HFOT) groups (DS: dyspnea score, HFOT: high flow oxygen therapy, SpO_2_: pulse oxymetry, pre and post: before and, respectively, after bronchoscopy, T0: time of beginning of bronchoscopy, T0.5, T1, T2: 30 s, 1 and 2 min, respectively, after the start of bronchoscopy).

Parameters	COT	HFOT	*p*-value
DS pre	2 (0; 3)	0 (0; 1.5)	0.37
DS post	0 (0; 2.5)	0.5 (0; 2.5)	0.92
Duration of tracheobronchoscopy (min)	2.5 (2; 5)	9.5 (3; 20.5)	0.04*
Duration of oxygen therapy (min)	29.5 (20; 39)	40 (21; 60)	0.38
SpO_2_ pre (%)	98 (± 2)	97 (± 1)	0.32
SpO_2_ post (%)	96 (± 3)	96 (± 2)	0.85
SpO_2_ T0 (%)	94 (± 2)	98 (± 2)	0.02*
SpO_2_ T0.5 (%)	94 (± 3)	98 (± 2)	0.02*
SpO_2_ T1 (%)	94 (± 4)	98 (±–2)	0.03*
SpO_2_ T2 (%)	91 (± 10)	97 (± 4)	0.21

Bold value and asterisk represent significant difference between both group.

**Figure 4:**
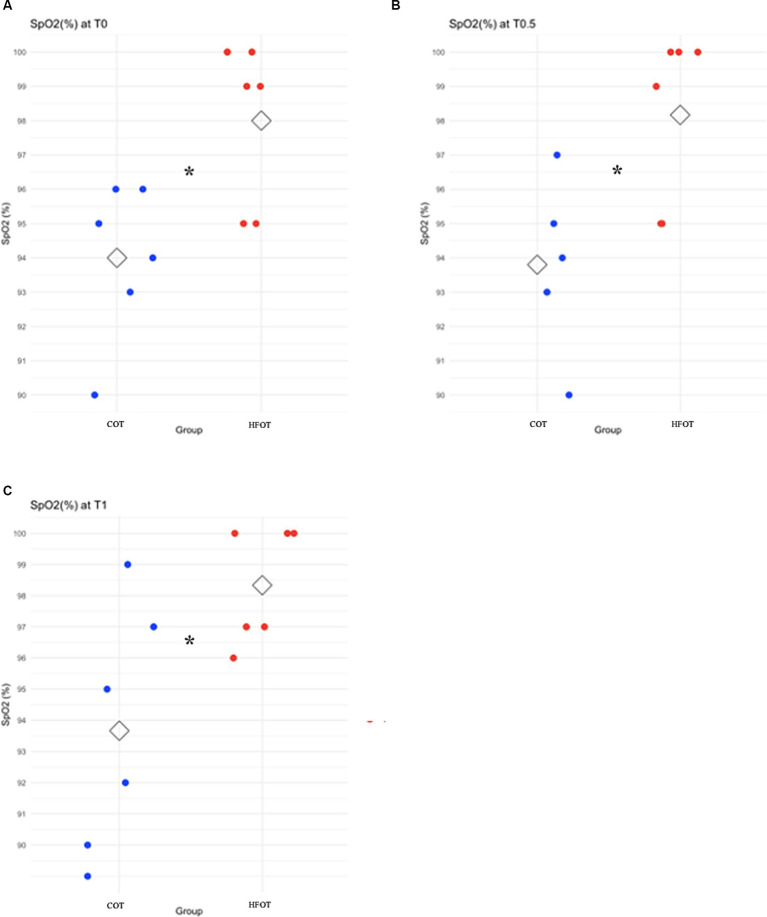
Scatter plot of pulse oxymetry (SpO_2_) in the conventional oxygen therapy (COT) group and the high flow oxygen therapy (HFOT) group at different time points during the bronchoscopy. The diamond represents the mean value for SpO_2_. Significant differences between groups are represented with an asterisk. **(A)** Significantly higher mean SpO_2_ for the HFOT group at T0 (*p* < 0.05), **(B)** Significantly higher mean SpO_2_ for the HFOT group at T0.5 (*p* < 0.05), **(C)** Significantly higher mean SpO_2_ for the HFOT group at T1 (*p* < 0.05).

Comparison of mean SpO_2_ at each time point revealed a significant difference between the two groups at the beginning of bronchoscopy. Mean SpO_2_ at T0, T0.5 and T1 was significantly higher in the HFOT group than in the COT group (98 (± 2) % vs. 94 (± 2) %, *p* = 0.02; 98 (± 2) % vs. 94 (± 3) %, *p* = 0.02; 98 (± 2) % vs. 94 (± 4) %, *p* = 0.04, respectively, Figure 4). Using a mixed model analysis, patients in the HFOT group had a significantly higher overall SpO_2_ during the bronchoscopy procedure compared with those in the COT group (*p* = 0.01, Figure 3).

There was no difference in the number of desaturation episodes between the groups (*p* = 0.45).

### 3.4 BAL procedure

Bronchoalveolar lavage was performed in six patients, including five in the HFOT group and one in the COT group. The median duration of BAL was 4.5 (2.5; 8) minutes. The median BAL yield was 50 (45; 60) %. No episode of severe desaturation occurred during fluid instillation.

## 4 Discussion

The results of this pilot study suggest that HFOT is safe and feasible in dogs and cats undergoing bronchoscopy, which is consistent with data from previous studies in dogs (4, 5). In our study, none of the patients that received HFOT experienced complications such as severe desaturation (SpO_2_ < 90%). In contrast, severe desaturation occurred in two patients in the COT group (one cat and one dog), necessitating interruption of the bronchoscopy procedure.

Our study included only three cats and there is currently no published information on the feasibility and safety of HFOT in a larger cat population. However, a recent case report describes good tolerance of nasal cannulas for 16 h, with no complications, in one cat with cardiogenic pulmonary edema treated with HFOT (19). This is consistent with our experience during this pilot study. We found that the technique was feasible in cats, as the nasal cannulas were subjectively easy to place in the nares of each patient, and did not appear to interfere with the bronchoscopic procedure.

Despite the overall feasibility of HFOT, bronchoscopy was significantly longer in the HFOT group compared with the COT group. While this could be a hazard error, as more patients in the HFOT group underwent BAL, the possibility that HFOT was associated with the increased duration of bronchoscopy could not be excluded. High flow oxygen therapy appeared to be safe in our pilot study, with no complications reported. These data are consistent with a recent study involving four dogs treated with HFOT during bronchoscopy. In that study, no adverse effects were observed, leading to the conclusion that HFOT may be a safe and well-tolerated alternative oxygen therapy technique during bronchoscopy (18). In the current study, thoracic and abdominal radiographs were not performed following bronchoscopy; hence, nonclinical pneumothorax or gastric dilatation may have gone undetected. Bronchoscopy and BAL are valuable tools in the evaluation of respiratory diseases in dogs and cats. However, these procedures necessitate general anesthesia and intubation is often not possible during the procedure due to the small size of the upper airway. Thus, HFOT may be of benefit in minimizing desaturation episodes. In fact, HFOT and constant positive airway pressure are the only oxygen therapy modalities that allow 100% FiO_2_ without intubation (1). Moreover, HFOT has many theoretical advantages, including increased FiO_2_, reduced dead space, and the generation of a small positive end-expiratory pressure that can prevent atelectasis and reduce upper airway resistance (1). However, these benefits are only expected in patients with closed-mouth breathing (20).

At our institution, bronchoscopies are performed with or without oxygen supplementation, depending on the underlying disease or clinical condition of the patient. If oxygen supplementation is required, it is delivered either through the working channel of the endoscope or, if the patient’s size permits, via an endotracheal tube with the endoscope passed through the tube using a T-adapter. As the latter is not feasible in small patients, we elected to administer oxygen through the working channel of the endoscope to all patients in the COT group. Conventional oxygen therapy can result in a maximum FiO_2_ of 40% (21). In contrast, HFOT can result in an FiO_2_ up to 100%. Therefore, patients in the HFOT group received a higher FiO_2_ than those in the COT group.

Hypoxemia is defined as PaO_2_ less than 80 mmHg. While arterial blood gas analysis is considered the gold standard for assessing oxygenation (22), SpO_2_ is a reliable surrogate for PaO_2_ in most hypoxemic and normoxemic patients. However, as demonstrated by the oxygen-hemoglobin dissociation curve, it is of little value in oxygen-supplemented hyperoxemic patients (PaO_2_ ≥ 100 mmHg) (23). A recent study confirmed that while SpO_2_ could be used to assess oxygenation in patients breathing room air, this was not the case in mechanically ventilated dogs, particularly in those with an SpO_2_ > 95% (24). Some studies have shown that the SpO_2_/FiO_2_ ratio can be used as a surrogate for the PaO_2_/FiO_2_ ratio to assess oxygenation in patients breathing room air or receiving nasal prong oxygenation (25, 26). Nevertheless, to our knowledge, there is no published data on the use of the SpO_2_/FiO_2_ and the PaO_2_/FiO_2_ ratio in dogs and cats undergoing HFOT. Despite these limitations, we chose to use SpO_2_ for several reasons. It allows continuous monitoring with a non-invasive technique (23) and is routinely used to monitor oxygenation during anesthesia. As our goal was to assess desaturation episodes, which can occur rapidly, a continuous measurement technique was essential.

We observed a higher overall SpO_2_ during bronchoscopy in the HFOT group compared to the COT group. The mean SpO_2_ before the procedure was not significantly different between both groups. This result was expected as many studies in veterinary and human medicine have shown an improvement in oxygen parameters in both dyspneic and hypoxemic patients receiving HFOT (2–7, 10, 12–17). Moreover, severe desaturation was not observed in any of the patients receiving HFOT, although some experienced mild desaturation episodes. Mean SpO_2_ was significantly higher in the HFOT group at T0, compared with the COT group, and this higher level of oxygenation prior to bronchoscopy may explain the absence of severe desaturation in the HFOT group. In a previous study, two of four dogs undergoing bronchoscopy with HFOT experienced desaturation episodes. One dog experienced severe desaturation, while the other experienced mild desaturation (18). In human medicine, the evidence for the benefits of HFOT during bronchoscopy is more robust. In a prospective randomized controlled trial involving 176 patients, those treated with HFOT had no severe desaturation episodes, defined as an SpO_2_ < 90% lasting for more than 60 s or SpO_2_ < 75%, and only a few (4/87) had moderate desaturation episodes, defined as SpO_2_ < 90% lasting for less than 60 s (11). Furthermore, a significantly higher incidence of oxygen desaturation was found in the facemask group compared with the HFOT group.

The current study revealed a low incidence of desaturation, with only one cat and one dog showing severe desaturation. This is in contrast to data from a retrospective multicenter study evaluating complications associated with bronchoscopy in cats (9). Severe desaturation with an SpO_2_ < 90% was the most common adverse event in that study, occurring in 24 of 79 cats. Due to the multicenter nature of this study, some variation was observed in the frequency of severe desaturation, which reached approximately 50% in one center (9). In another study, complications (ranging from mild to life-threatening) occurred in 26 of 68 enrolled cats. Of the 26 cats, 16 experienced mild complications defined as a decrease in SpO_2_ during the procedure and/or immediate termination of the procedure. However, four of the 26 cats experienced moderate complications, two cats suffered severe complications requiring intensive care recovery, and four cats were euthanized after the procedure (27). These data are consistent with the fact that two thirds of the cats included in the current study were assessed as having a high risk of desaturation episodes. To our knowledge, there is no data on the frequency of desaturation episodes during bronchoscopy in dogs.

Our pilot study had several limitations. First, given the small enrolled population, severe desaturation in one group could have significantly impacted the accuracy of the SpO_2_ analysis. Although patients were randomized, the randomization process did not take into account species, respiratory diseases and their effects on respiratory function and BAL performance. The heterogeneity of the population, which comprised both dogs and cats, with different risks of desaturation episodes during anesthesia and bronchoscopy, is another limitation. Second, the HFOT group included more animals suffering from bronchial or bronchopulmonary diseases, including two cats considered to be at high risk for desaturation episodes. On the contrary, all but one of the animals in the COT group were considered to be at normal risk of desaturation and suffered from upper airway disease. Third, in this study, only one patient in the COT group underwent BAL, compared with five in the HFOT group. This highlights the limitations of randomization in a small sample population for creating equal groups. However, a retrospective study of bronchoscopy and BAL complications in cats reported that disease and BAL characteristics (total volume of lavage fluid instilled, volume per kg used, number of BAL aliquots instilled, or percent fluid recovery) were not associated with complications (27). Thus, while we acknowledge that our two study groups were not equivalent, the major biases appear to increase the likehood of more frequent and/or more severe desaturation episodes within the HFOT group. Nevertheless, we found a significantly higher overall SpO_2_ in the HFOT group compared with the COT group, highlighting the potential beneficial effects of HFOT. It is important to note that the two groups received different FiO_2_ due to the nature of the oxygenation techniques, which may have led to a bias in favor of HFOT. Finally, finding a difference between two groups was not part of the design of this pilot study. Therefore, a larger study with a more homogeneous population is needed to confirm our results.

## 5 Conclusion

To the authors’ knowledge, this is the largest study conducted to date using HFOT during bronchoscopy in dogs and cats. High flow oxygen therapy was feasible and easy to set up during bronchoscopy and was not associated with complications. The patients that received HFOT had no severe desaturation episodes (SpO_2_ < 90%), suggesting that HFOT may limit desaturation episodes, notably in hypoxemic patients, during bronchoscopy and BAL.

## Data availability statement

The raw data supporting the conclusions of this article will be made available by the authors, without undue reservation.

## Ethics statement

The animal studies were approved by ethic committee of VetAgro Sup. The studies were conducted in accordance with the local legislation and institutional requirements. Written informed consent was obtained from the owners for the participation of their animals in this study.

## Author contributions

CD: Conceptualization, Data curation, Formal analysis, Investigation, Methodology, Validation, Writing – original draft, Writing – review & editing. MA: Data curation, Writing – review & editing. SJ: Conceptualization, Methodology, Supervision, Validation, Writing – review & editing. AN: Supervision, Validation, Writing – review & editing. BA: Conceptualization, Formal analysis, Supervision, Validation, Writing – review & editing. EK: Conceptualization, Methodology, Supervision, Validation, Writing – review & editing. CP-N: Conceptualization, Data curation, Investigation, Methodology, Project administration, Supervision, Validation, Writing – review & editing.
